# Postoperative adjuvant immunotherapy and molecular targeted therapy for patients of hepatocellular carcinoma with portal vein tumor thrombus after hepatectomy: a propensity score matching study

**DOI:** 10.3389/fsurg.2024.1387246

**Published:** 2024-08-07

**Authors:** Jiangmin Zhou, Huifang Xiong, Zhiwei Zhang, Dong Chen, Wei Wang, Cheng Zhou, Biao Wu

**Affiliations:** ^1^ Department of Hepatobiliary Surgery, Wuhan No.1 Hospital (Wuhan Hospital of Traditional Chinese and Western Medicine), Wuhan, China; ^2^ Department of Digestive Internal Medicine, Wuhan Dongxihu District People Hospital, Wuhan, China; ^3^ Department of Hepatic Surgery, Tongji Hospital, Tongji Medical College, Huazhong University of Science and Technology, Wuhan, China

**Keywords:** hepatocellular carcinoma, portal vein tumor thrombus, postoperative adjuvant, propensity score matching study, immunotherapy combined therapy

## Abstract

**Background:**

Portal vein tumor thrombus (PVTT) is a major risk factor of recurrence of hepatocellular carcinoma (HCC) after hepatectomy. Whether postoperative adjuvant immunotherapy and molecular targeted therapy (I-O and MTT) is effective in reducing the risk of recurrence of HCC with minimal portal invasion after hepatectomy and improving prognosis is unknown.

**Methods:**

We collected the data of HCC with Vp1 or Vp2 PVTT patients who underwent hepatectomy at our center between January 2019 and June 2022 from the hospital database. We utilized propensity score matching (PSM) to establish a 1:1 match between the postoperative group treated with I-O and MTT and the postoperative group without I-O and MTT. To compare the recurrence-free survival (RFS) and overall survival (OS) between the two groups, we employed the Kaplan-Meier method. Additionally, we conducted Cox regression analysis to identify the prognostic factors that influence patient prognosis. To account for different high-risk factors, subgroup analyses were carried out.

**Results:**

Among the 189 patients included in the study, 42 patients received postoperative adjuvant I-O and MTT. After PSM, the 1, 2-years RFS were 59.2%, 21.3% respectively in the I-O and MTT group and 40.8%, 9.6% respectively in the non-I-O and MTT group. The median RFS was 13.2 months for the I-O and MTT group better than 7.0 months for the non-I-O and MTT group (*P* = 0.028). 1, 2-years OS were 89.8%, 65.8% respectively in the I-O and MTT group and 42.4%, 27.7% respectively in the non-I-O and MTT group. The median OS was 23.5 months for the I-O and MTT group better than 17.2 months for the non-I-O and MTT group (*P* = 0.027). Multivariate analysis showed that postoperative adjuvant I-O and MTT was a prognostic protective factor associated with OS and RFS. The most frequent AE observed in this study was pruritus, and rare AEs included decreased platelet, hypothyroidism, proteinuria, myocarditis and hypoadrenocorticism. The incidence of GRADE ≥3 AE with no deaths recorded.

**Conclusion:**

The study suggested that postoperative adjuvant I-O and MTT strategy was beneficial to improve the prognosis of HCC patients with PVTT patients, while the therapy was safe and reliable.

## Introduction

Hepatocellular carcinoma (HCC) is a common cancer with a poor prognosis. Hepatectomy, an aggressive surgical procedure, is frequently employed as the primary approach for eradicating HCC. However, its efficacy is compromised by a substantial tumor recurrence rate (70%) occurring within 5 years post-surgery (1). This is particularly evident in patients possessing high-risk factors for recurrence, including microvascular invasion, portal vein tumor thrombosis, as well as multiple tumor nodules. It is now generally accepted that the early spread of tumor cells through the bloodstream, especially in HCC with portal vein tumor thrombus (PVTT), is a key mechanism for intrahepatic metastasis and tumor recurrence (2, 3). A previous study reported that only 10% of patients with tumor thrombi in the first branch and portal trunk survived more than 5 years following hepatectomy (4). Unfortunately, there is currently few recommended postoperative treatment strategies for HCC patients with PVTT, which poses a major challenge in managing these patients. As such, it is imperative to provide HCC patients with effective adjuvant treatments following liver resection in order to mitigate recurrence and enhance long-term survival rates.

Fortunately, significant progress has been made in the treatment of unresectable HCC with the use of immune checkpoint inhibitors (ICIs) (5–7). In the case of certain types of tumors like melanoma, esophageal cancer, and gastric cancer, the effectiveness of anti-programmed death 1 (PD-1) antibodies in prolonging patients' overall survival (OS) and recurrence-free survival (RFS) has been proven. As a result, the mechanisms of ICIs offer a promising approach for postoperative adjuvant therapy in HCC patients. Ongoing phase III clinical trials (8, 9) currently indicate the potential of postoperative PD-1 antibodies in effectively extending the survival of patients at high risk of postoperative recurrence.

The question being addressed in this study is whether postoperative adjuvant therapy with I-O and MTT can reduce the risk of postoperative recurrence in HCC patients who have undergone liver resection and have high-risk factors for recurrence. Therefore, this retrospective study was designed to assess the effectiveness of I-O and MTT for HCC patients with PVTT after hepatectomy.

## Materials and methods

From January 2019 to June 2022, the data for this study were gathered from patients who had undergone curative hepatic resection at Tongji Medical College of Huazhong University of Science and Technology. We enforced strict criteria to include or exclude patients. The inclusion criteria encompassed the following: (1) individuals aged over 18, (2) a postoperative diagnosis of HCC confirmed through pathology, (3) liver resection and thrombectomy, (4) initial detection of the tumor, (5) being classified as Child-Pugh class A or B, and (6) having an Eastern Cooperative Oncology Group (ECOG) performance status between 0 and 1. On the other hand, patients were excluded if they met any of the following criteria: (1) a history of prior anticancer treatment, (2) incomplete records for follow-up, or (3) failure to comply with drug therapy, changing scheme midway, such as Sorafenib, Bevacizumab, Atezolizumab, etc. The study was approved by the Ethical Committee of Tongji Medical College of Huazhong University of Science and Technology [TJ-IRB20191101], and all procedures followed the Declaration of Helsinki. Informed consent was obtained from all the patients included in the study.

Portal vein tumor thrombosis is characterized by the presence of tumor emboli within the portal vein. PVTT can be classified into two types: Type I (Vp1), which involves the invasion of third-order portal vein branches, and Type II (Vp2), which involves the invasion of second-order portal vein branches (10). The evaluation of PVTT could be conducted using contrast-enhanced CT or MRI. To assess the overall condition of patients, the ECOG performance status was utilized. before surgery, abdominal CT and MRI are performed for preoperative assessments. In order to determine liver functional reserve, the clearance of Indocyanine Green at 15 min (ICG-15) was measured. Intraoperative ultrasound was then conducted to evaluate the incisal margin and assess the removal of PVTT. The hepatectomy procedure, either open or laparoscopic, is carried out by a professional team along with thrombectomy. All patients included in the study had sufficient liver function reserve and underwent either major (resection of three or more liver segments) or minor (resection of one or two liver segments) hepatectomy.

### Usage of I-O and MTT

On the day of discharge, patients started receiving postoperative adjuvant treatment. Both Lenvatinib and Pembrolizumab were used in I-O and MTT scheme. This included oral administration of Lenvatinib (Lenvima®, Eisai, Tokyo, Japan) at a dose of either 8 mg/day for patients weighing less than 60 kg or 12 mg/day for those weighing 60 kg or more (11). Additionally, Pembrolizumab (KEYTRUDA, Merck Sharp & Dohme Co., Inc.) was administered at a dose of 200 mg per infusion, every 3 weeks, intravenously (12). The dose and duration of Pembrolizumab were determined according to the guidelines or expert consensus. All patients in the treatment group completed I-O and MTT therapy, and adverse events occurred in all patients, including 10 patients with grade 3 or above adverse events. After adjustment of drug dose and treatment frequency, all patients completed the whole treatment.

### Follow-up and tumor recurrence

**Figure 1:**
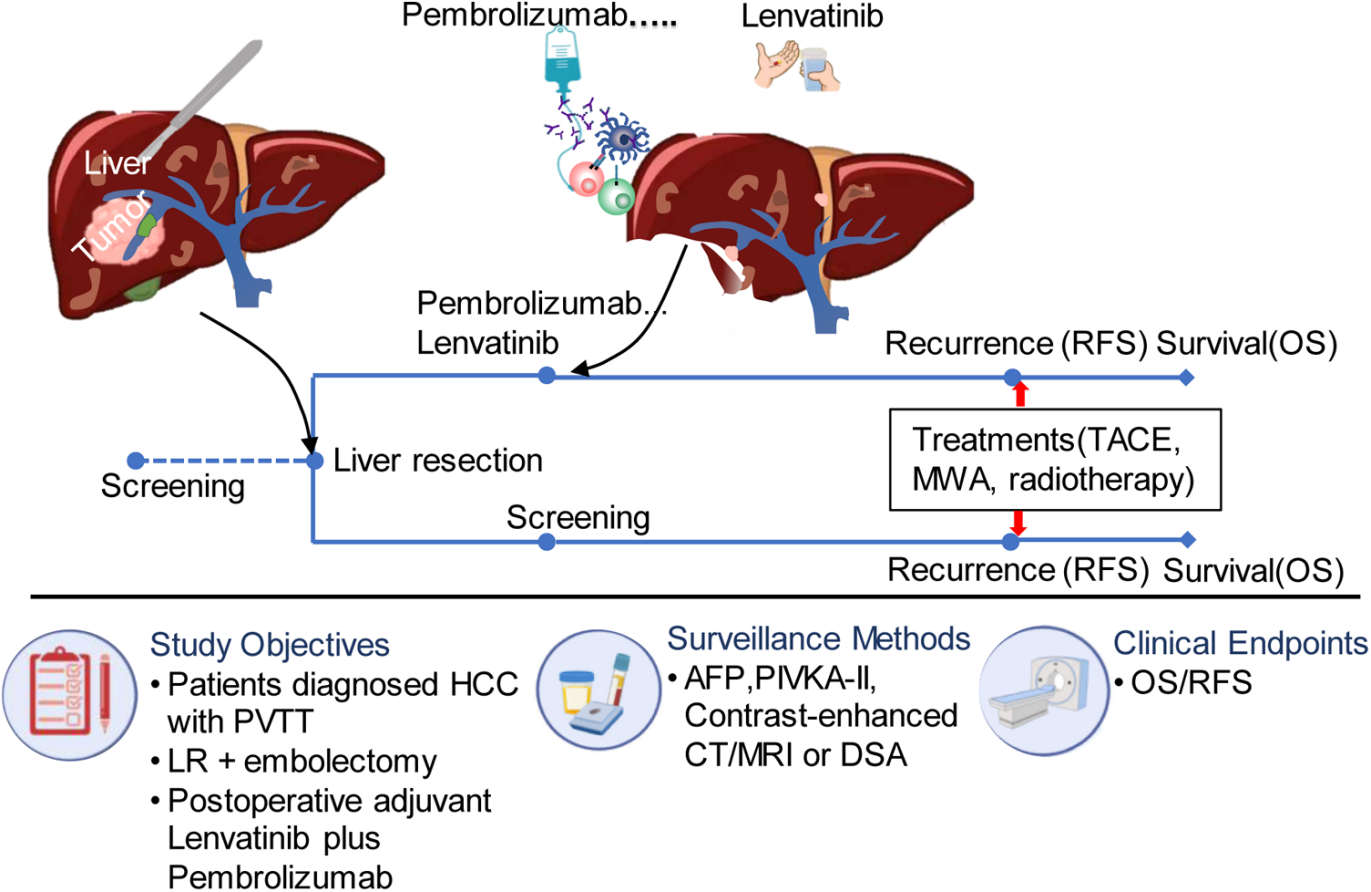
The flow diagram of treatment for eligible patients. PVTT, portal vein tumor thrombus; TACE, transcatheter arterial chemoembolization; MWA, microwave ablation; AFP, alpha fetoprotein; HCC, hepatocellular carcinoma; DSA, digital subtraction angiography; RFS, recurrence free survival; PIVKA, vitamin K deficiency or antagonist; LR, liver resection.

The postoperative surveillance included monthly ultrasonography and measurements of serum alpha-fetoprotein (AFP) and protein induced by vitamin K absence or antagonist (PIVKA-II) levels for the first six months after surgery. After that, measurements were taken every three months. Recurrence was definitively diagnosed through contrast-enhanced CT or MRI examinations, and tumor progression was evaluated. Patients with recurrence received locoregional therapy such as transcatheter arterial chemoembolization (TACE), microwave ablation, or locoregional radiotherapy. The detailed flow scheme can be seen in Figure 1. Follow-up was concluded on April 24, 2023. The patient's survival status and the potential drug-related toxicities was determined through governmental death registration and telephone follow-up.

### Statistics analysis

Statistical analysis was performed using IBM SPSS Statistics version 22 (SPSS Inc., Chicago, USA) and R 4.0.2 (http://www.R-project.org). Continuous variables were presented as the median and interquartile range (IQR) and categorical data as number and percentage. *χ*^2^ test or Mann-Whitney *U*-test was used for comparison between groups where appropriate. To account for confounding variables among the two groups, we carried out propensity score matching (PSM) in a 1:1 ratio. Propensity scores were derived using binary logistic regression with chosen variables and represented as continuous values ranging from 0 to 1. For matching patients in the I-O and MTT group with those in the non- I-O and MTT group, we applied nearest-neighbor matching. The relationship between the prognosis and different treatment strategies was analyzed using Kaplan-Meier survival curves and a log-rank test. We divided all patients into the training set (*n* = 147) and the validation set (*n* = 42). Univariate and multivariate Cox proportional regression analysis were used to evaluate risk factors for recurrence or survival. A nomogram for predicting prognosis was established based on the results of the multivariate analysis. The predictive accuracy of the nomogram was assessed by calibration. Receiver operating characteristic curve (ROC) was used to evaluate the predictive value of the independent risk factors for survival. In addition, in order to evaluate the performance of diagnostic tests and prediction models, decision curve analysis (DCA) was included in the study. DCA serves as a tool to assess the effectiveness of prediction models in clinical decision-making. A common scenario for DCA application is when patients exhibit symptoms indicative of a potential disease, but a definitive diagnosis has not yet been made. Clinicians face the challenge of deciding whether to proceed with a biopsy or screening procedure to confirm the disease (13–15). A two-sided *P* < 0.05 was considered statistically significant.

## Results

### Patient demographic and baseline clinical characteristics

Our institution employed rigorous inclusion and exclusion criteria to select a collective of 189 individuals who underwent liver resection and thrombectomy in the Hepatic Surgery between January 2019 and June 2022. Among these patients, a subset of 42 individuals underwent postoperative treatment with I-O and MTT. There were 40 males, accounting for 95.2% of the validation set. 83.3% of patients were infected chronic hepatitis B virus infection (35/42). Forty patients were classified as Child-Pugh class A, indicating well-preserved liver function, and 35 patients (83.3%) had an ECOG performance status of 0 at baseline. Baseline differences existed in several variables between the two groups, including sex, AFP, PIVKA-II, tumor size, tumor number, MVI, satellite lesion and tumor capsule, while the other variables showed no statistical differences. To balance these baseline differences, a 1:1 PSM was performed on the two groups of patients. Following PSM, there were no statistical differences in any variables between the two groups. Detailed data distribution could be found in Table 1. The mean follow-up time was 30.5 months (median, 31.5 months; range, 15.6–51.2 months).

**Table 1:** Patients baseline clinical and pathological characteristics.

Clinical characteristics	Before PSM			After PSM		
	Validation set (*N* = 42)	Training set (*N* = 147)	*P*	Validation set (*N* = 42)	Training set (*N* = 42)	*P*
Age (years)						
Median (IQR)	50 (41–56)	53 (42–58)	0.074	50 (41–56)	51 (40–57)	0.098
Sex, *N* (%)						
Male	40 (95.2)	119 (81.0)	0.016^a^	40 (95.2)	37 (88.1)	0.433
Female	2 (4.8)	28 (19.0)		2 (4.8)	5 (11.9)	
Child-Pugh score, *N* (%)						
A	40 (95.2)	128 (87.1)	0.171	40 (95.2)	39 (92.9)	1.000
B	2 (4.8)	19 (12.9)		2 (4.8)	3 (7.1)	
ECOG performance status, *N* (%)						
0	35 (83.3)	120 (81.7)	0.800	35 (83.3)	32 (76.2)	0.415
1	7 (16.7)	27 (18.3)		7 (16.7)	10 (23.8)	
Cause of disease, *N* (%)						
Hepatitis B	35 (83.3)	117 (79.6)	0.804	35 (83.3)	31 (73.8)	0.445
Hepatitis C	2 (4.8)	11 (7.5)		2 (4.8)	5 (11.9)	
Other	5 (11.9)	19 (12.9)		5 (11.9)	6 (14.3)	
Cirrhosis, *N* (%)						
Yes	36 (85.7)	128 (87.1)	0.818	36 (85.7)	40 (95.2)	0.625
No	6 (14.3)	19 (12.9)		6 (14.3)	2 (4.8)	
ALT (U/L)						
Median (IQR)	31 (24–102)	33 (25–98)	0.124	31 (24–102)	34 (28,112)	0.298
Albumin						
Median (IQR)	39.4 (34.2–41.0)	38.0 (32.1–40.2)	0.254	39.4 (34.2–41.0)	38.2 (31.8–39.8)	0.587
Total bilirubin						
Median (IQR)	14.5 (12.4–31.2)	11.6 (10.2–29.3)	0.369	14.5 (12.4–31.2)	13.5 (12.4–30.8)	0.985
AFP (ng/ml)						
Median (IQR)	214 (17.9–1,534)	178 (15.3–1,020)	0.027	214 (17.9–1,534)	201 (14.3–1,029)	0.484
PIVKA-II (mAU/ml)						
Median (IQR)	416 (38–2,894)	267 (32–2,140)	0.011	416 (38–2,894)	389 (40–2,598)	0.698
Edmondson-Steiner stage						
I–II	14 (33.3)	55 (37.4)	0.628	14 (33.3)	18 (42.9)	0.501
III–IV	28 (66.7)	92 (62.6)		28 (66.7)	24 (57.1)	
Tumor size (cm)						
Median (IQR)	6.9 (4.7–9.9)	5.0 (4.0–8.5)	0.038	6.9 (4.7–9.9)	6.6 (4.4–8.9)	0.214
Tumor number						
Single	26 (61.9)	117 (79.6)	0.018	26 (61.9)	29 (69.0)	0.126
Multiple	16 (38.1)	30 (20.4)		16 (38.1)	13 (31.0)	
MVI						
Positive	31 (73.8)	81 (55.1)	0.030	31 (73.8)	29 (69.0)	0.629
Negative	11 (26.2)	66 (44.9)		11 (26.2)	13 (31.0)	
Satellite lesion						
Positive	17 (40.5)	32 (21.8)	0.015	17 (40.5)	16 (38.1)	0.823
Negative	25 (59.5)	115 (78.2)		25 (59.5)	26 (61.9)	
Tumor capsule						
Complete	10 (23.8)	68 (46.3)	0.009	10 (23.8)	14 (33.3)	0.469
Incomplete	32 (76.2)	79 (53.7)		32 (76.2)	28 (66.7)	
Surgery method						
Open	35 (83.3)	110 (74.8)	0.250	35 (83.3)	31 (73.8)	0.287
Laparoscopic	7 (16.7)	37 (25.2)		7 (16.7)	11 (26.2)	

PSM, propensity score matching; ECOG, Eastern Cooperative Oncology Group; ALT, alanine transaminase; PLT, platelet; AFP, alpha-fetoprotein; PIVKA, protein induced by vitamin K absence or antagonist; IQR, interquartile range; MVI, microvascular invasion.

^a^ Fisher's precision probability test.

At the end of follow-up, 168 of 189 patients relapsed, the recurrence rate was 88.9%, and 135 patients died, the mortality rate was 71.4%. Of the 168 patients with recurrence, 121 had only intrahepatic recurrence, 30 had lung metastasis, and the rest had multiple extrahepatic metastases. All relapsed patients received local treatment, including 78 patients who received TACE alone, 48 patients who received microwave ablation (MWA) alone, and 42 patients who received TACE/MWA combined with radiotherapy.

In addition, 8 patients in the training set received I-O and MTT at the time of postoperative recurrence, while other patients with recurrence received only local treatment.

### Survival

**Figure 2:**
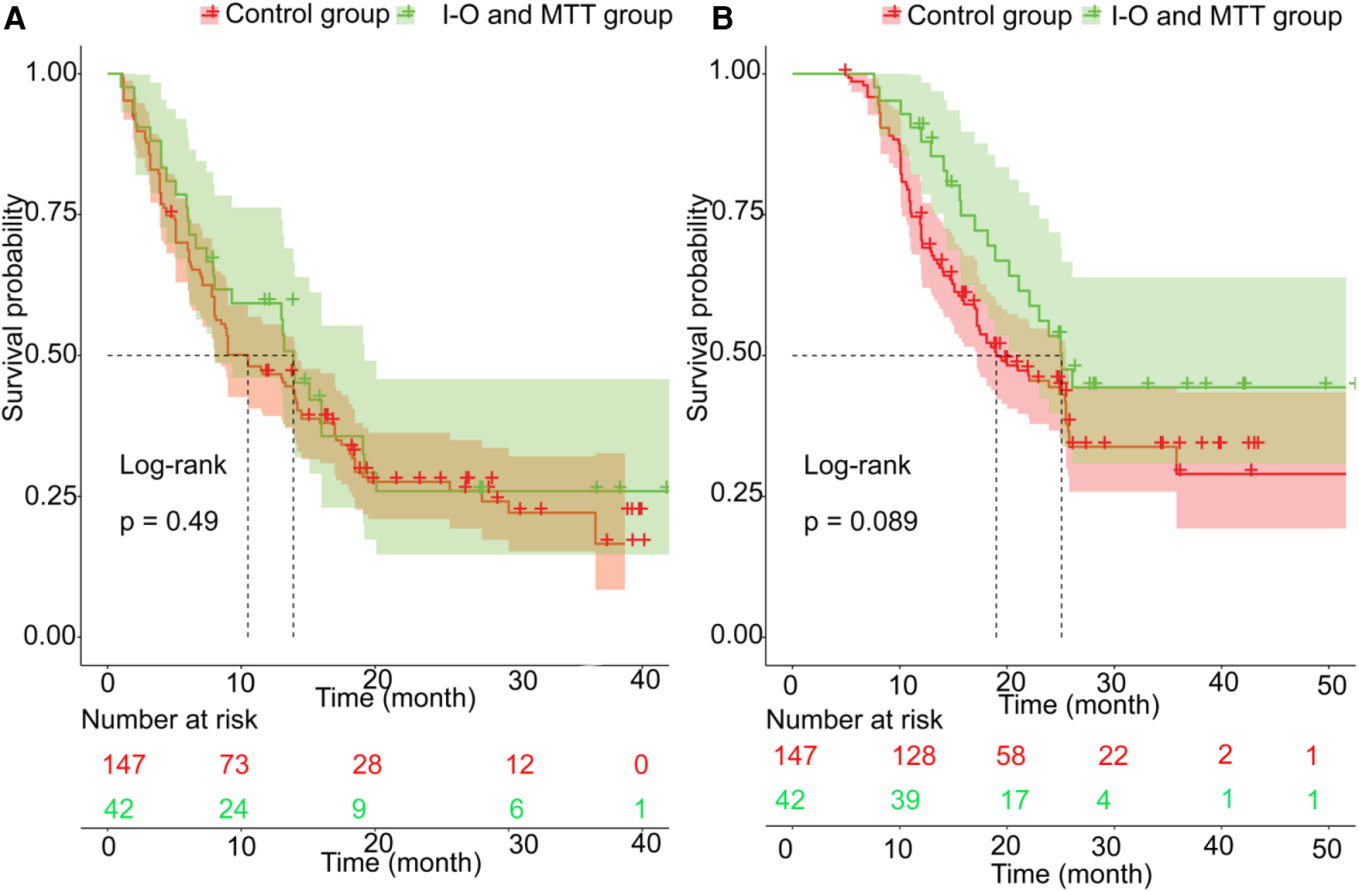
The prognosis comparison. between validation set and training set before PSM; (**A**) the cumulative recurrence rate comparison between two groups; (**B**) the cumulative survival rate comparison between two groups.

**Figure 3:**
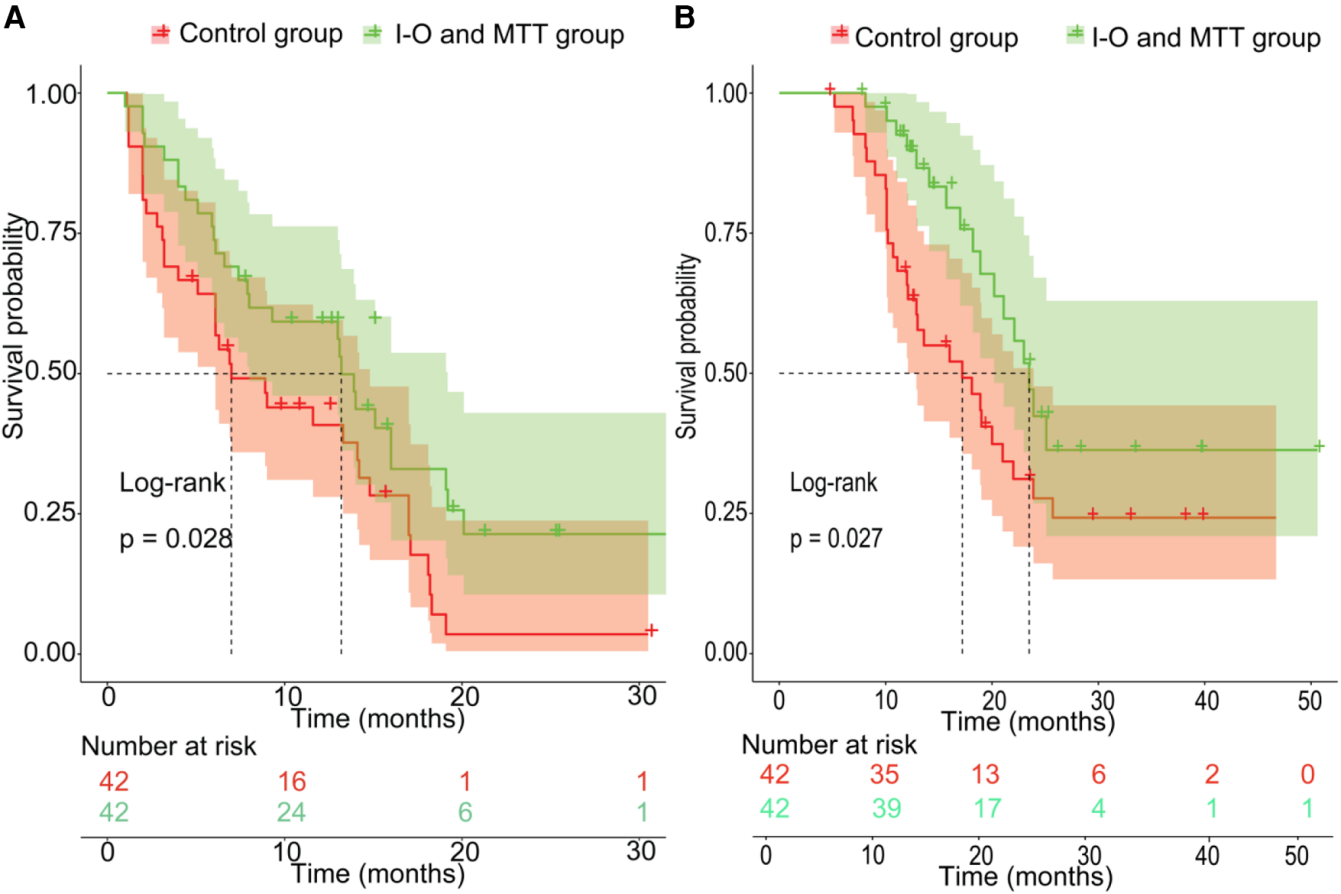
The prognosis comparison between validation set and non-I-O and MTT group after PSM; (**A**) the cumulative recurrence rate comparison between two groups; (**B**) the cumulative survival rate comparison between two groups.

Before PSM, the patients in the validation set had 1-year, 2-year recurrence free survival rates of 59.3% and 25.9%, respectively. In contrast, the patients in the training set had 1-year and 2-year recurrence free survival rates of 46.7% and 27.6%, respectively. There was no statistical difference between the two groups (*P* = 0.49) (Figure 2A). Regarding OS, before PSM, the validation set had 1-year and 2-year OS rates of 88.0% and 53.5%, respectively, while the training set had 1-year and 2-year OS rates of 70.5% and 44.4%, respectively. There was no statistical difference between the two groups (*P* = 0.089) (Figure 2B). After PSM, the patients in the validation set had the 1-year and 2-year recurrence free survival rates were 59.3% and 25.9%, respectively, compared to 40.8% and 9.6%, respectively, in the training set. The median RFS was 13.2 months (95% CI 8.0–19.2 months) for the validation set, which was better than the training set with a median RFS of 7.0 months (95% CI 6.1–14.8 months) (*P* = 0.028) (Figure 3A). The 1-year and 2-year overall survival rates were 88.0% and 53.5%, respectively, in the validation set, compared to 42.4% and 27.7%, respectively, in the training set. The median OS was 23.5 months (95% CI 20.2–29.0 months) for the validation set, which was better than the training set with a median OS of 17.2 months (95% CI 12.9–23.9 months) (*P* = 0.027) (Figure 3B).

### Independent prognostic factors of HCC with PVTT

Multivariate Cox proportional hazards regression analysis was conducted to identify independent risk factors for recurrence and overall survival after hepatectomy for HCC with PVTT. The analysis revealed that AFP >400 ng/ml [*P* = 0.009, hazard ratio (HR) = 5.452 (4.025–7.895)], PIVKA-II >40 mAU/ml [*P* = 0.041, HR = 2.251 (1.123–4.542)], incomplete tumor capsule [*P* = 0.013, HR = 4.279 (4.124–5.403)], Edmondson-Steiner grade III–IV [*P* = 0.019, HR = 3.547 (1.963–4.254)], and I-O and MTT [*P* = 0.042, HR = 0.657 (0.352–0.821)] were identified as independent risk factors for recurrence. Similarly, AFP >400 ng/ml [*P* = 0.012, HR = 2.125 (1.874–4.356)], incomplete tumor capsule [*P* = 0.032, HR = 3.387 (1.811–4.474)] and I-O and MTT [*P* = 0.037, HR = 0.851 (0.622–0.914)] were independent prognostic factors of overall survival after hepatectomy for HCC with PVTT (Tables 2, 3).

**Table 2:** Univariate regression analysis of recurrence free survival and overall survival.

Variables	RFS		OS	
	HR (95% CI)	*P*	HR (95% CI)	*P*
Age, >60 years	1.218 (0.423–1.574)	0.258	1.102 (0.941–1.195)	0.269
Sex, male	1.178 (0.941–1.224)	0.547	1.184 (0.954–1.215)	0.147
Child-Pugh, B	1.196 (0.947–1.264)	0.158	1.241 (1.023–1.598)	**0.029**
HBsAg, positive	1.211 (0.987–1.348)	0.215	1.354 (0.921–1.547)	0.147
Antiviral therapy, yes	0.754 (0.623–1.158)	0.369	0.874 (0.748–1.028)	0.258
AFP, >400 ng/ml	1.657 (1.451–1.982)	**0.024**	1.857 (1.259–2.547)	**0.021**
PIVKA-II, > 40 mAU/ml	1.251 (1.123–1.542)	**0.041**	1.236 (1.034–1.752)	**0.038**
Total bilirubin, >17.1 umol/L	1.222 (0.975–1.357)	0.214	1.025 (0.987–1.125)	0.657
Albumin, <35 g/L	1.023 (0.961–1.247)	0.146	1.128 (0.914–1.245)	0.269
PLT, <100 × 10^9^/L	1.297 (0.894–1.368)	0.249	1.024 (0.958–1.256)	0.143
Liver cirrhosis, yes	1.052 (0.899–1.205)	0.248	1.057 (0.854–1.112)	0.259
Tumor capsule, incomplete	1.874 (1.258–2.548)	**0.026**	2.416 (1.698–4.121)	**0.016**
Tumor size, >5 cm	1.279 (0.924–1.403)	0.213	1.279 (0.879–1.403)	0.104
Satellite lesions, yes	2.147 (1.879–3.654)	**0.022**	3.261 (2.159–6.298)	**0.017**
MVI, yes	1.198 (0.958–1.362)	0.071	1.142 (0.947–1.324)	0.109
Edmondson-Steiner grade, III–IV	1.369 (1.025–1.754)	**0.021**	1.142 (0.921–1.259)	0.143
I-O and MTT, yes	0.6572 (0.552–0.914)	**0.042**	0.778 (0.644–0.974)	**0.034**

Bold represents a *P* value <0.05, and the difference is statistically significant.

**Table 3:** Multivariate regression analysis of recurrence free survival and overall survival.

Variables	RFS		OS	
	HR (95% CI)	*P*	HR (95% CI)	*P*
AFP, >400 ng/ml	5.452 (4.025–7.895)	**0.009**	2.125 (1.874–4.356)	**0.012**
PIVKA-II, >40 mAU/ml	2.251 (1.123–4.542)	**0.041**	1.568 (0.951–1.687)	0.225
Tumor capsule, incomplete	4.279 (4.124–5.403)	**0.013**	3.387 (1.811–4.474)	**0.032**
Satellite lesions, yes	1.587 (0.856–1.923)	0.189	1.352 (0.741–1.658)	0.114
Edmondson-Steiner grade, III–IV	3.547 (1.963–4.254)	**0.019**	1.987 (0.951–2.871)	0.289
I-O and MTT, yes	0.657 (0.352–0.914)	**0.042**	0.851 (0.622–0.914)	**0.037**

HR, hazard ratio; OS, overall survival; RFS, recurrence free survival; CI, confidence interval; HBsAg, hepatitis B surface antigen; AFP, alpha-fetoprotein; PIVKA, protein induced by vitamin K absence or antagonist; PLT, platelet; MVI, microvascular invasion; I-O and MTT, immunotherapy and molecular targeted therapy.

Bold represents a *P* value <0.05, and the difference is statistically significant.

### Nomogram model of HCC with PVTT

**Figure 4:**
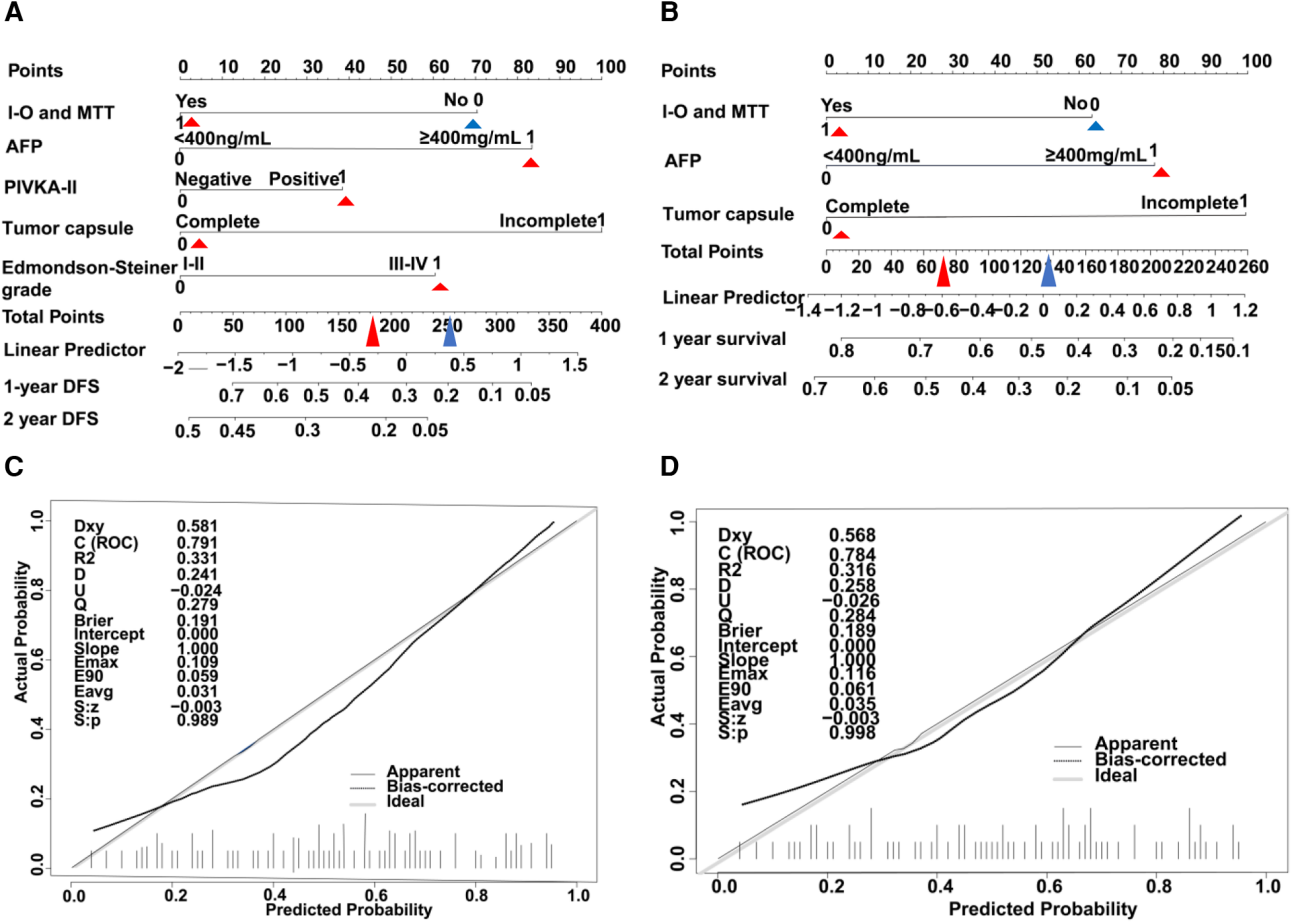
HCC with PVTT nomograms and calibration curves in training set. The nomogram RFS (**A**) and the nomogram OS (**B**) were constructed for evaluating prognosis of HCC with PVTT, respectively. Each variable is assigned a point on the top axis by drawing a line upward. The sum of these numbers is located on the total points axis, and a line is drawn downwards to the probability axis to identify the likelihood of prognosis of HCC with PVTT. The calibration curves for predicting RFS (**C**) and OS (**D**) in HCC with PVTT patients. Nomograms-predicted probability of prognosis is plotted on the x-axis, and actual probability is plotted on the y-axis.

**Figure 5:**
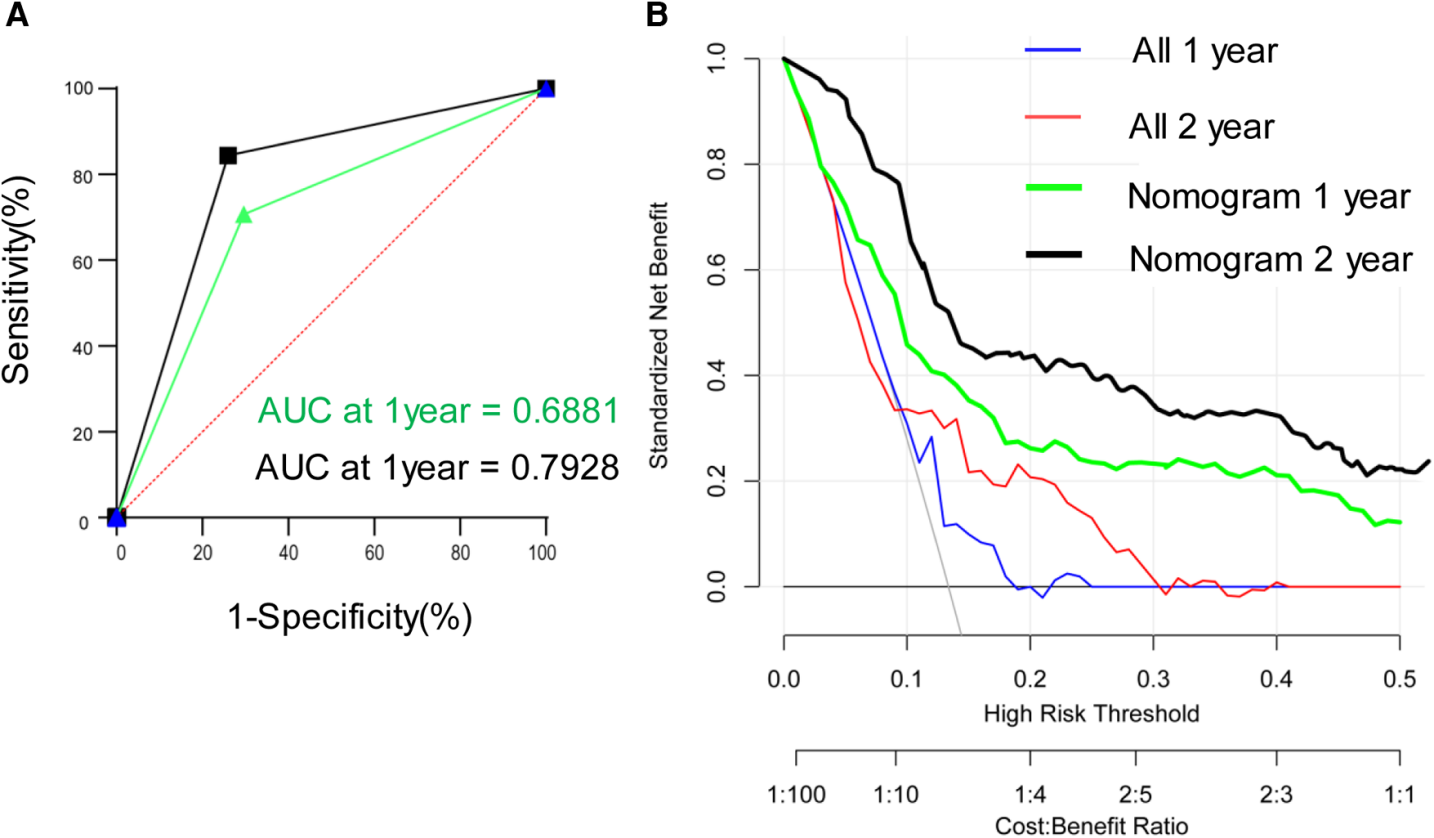
Time-dependent receiver operating characteristic curves for validation set (**A**) and decision curve analyses (**B**) in the validation set.

A nomogram model was developed to predict the recurrence risk of HCC with PVTT, incorporating important predictors identified in the Cox analysis. For instance, a patient with I-O and MTT (0 points) had an AFP >400 ng/ml (83 points), PIVKA-II >4 0mAU/ml (39 points), complete tumor capsule (0 points), and Edmondson-Steiner grade III-IV (60 points). The total score for this patient is 182 points, with an estimated 1-year recurrence-free survival rate of approximately 36% and a 2-year recurrence-free survival rate of approximately 23% (red triangle in Figure 4A). Additionally, the total score is 77 points, resulting in an estimated 1-year overall survival rate of about 66% and a 2-year overall survival rate of about 45% (red triangle in Figure 4B). In contrast, if the patient did not receive I-O and MTT (70 points), the total score would be 252 points. In this scenario, the estimated 1-year recurrence-free survival rate would be approximately 21% and the 2-year recurrence-free survival rate would be approximately 5% (blue triangle in Figure 4A). Furthermore, the total score would be 137 points, leading to an estimated 1-year overall survival rate of about 47% and a 2-year overall survival rate of about 23% (blue triangle in Figure 4B). Internal verification demonstrated that the nomogram accurately predicted the C-index of RFS and OS with values of 0.791 and 0.784, respectively. The calibration plot for the probability of prognosis indicated excellent agreement between the predictions and the actual observations (Figures 4C,D). 1-year and 2-year survival rates were included in the ROC curve. We found that the area under the curve (AUC) were 0.6881 (95% CI 0.5658–0.8103) and 0.7928 (95% CI 0.6818–0.9038) respectively (Figure 5A). In addition, in the DCA (Figure 5B), this model also shows good prediction potential.

### The incidence of adverse events (AEs)

The incidence of adverse events in I-O and MTT group was 100% (42/42). Table 4 summarized the observed representative AEs. Pruritus (95.2%) and fatigue (90.5%) were the most common adverse events in most patients, but no adverse events above grade 3 occurred. Elevated transaminase (>3*upper limit of normal) was a common adverse event observed in 25 (59.5%) patients, followed by diarrhea in 21 (50%) patients, rash in 18 (42.9%) patients, decreased platelet in 12 (28.6%) patients, cutaneous toxic effects in 11 (26.2%) patients, hypothyroidism in 5 (11.9%) patients and proteinuria in 4 (9.5%) patients respectively. Relatively rare adverse events were myocarditis in one (2.4%) patient and hypoadrenocorticism in one (2.4%) patient. The pruritus was the earliest AEs and median (IQR) was 4.1 weeks (3.2–8.5), followed by rash with 5.0 weeks (3.1–8.5), fatigue with 5.2 weeks (3.6–8.2), cutaneous toxic effects with 5.5 weeks (3.4–9.2), proteinuria with 6.3 weeks (4.5–10.2), elevated transaminase with 6.6weeks (3.9–7.9), diarrhea with 8.2 weeks (3.8–13.2) and decreased platelet 10.8weeks (6.3–15.2) respectively.

**Table 4:** Incidence of treatment-related adverse events in I-O and MTT group.

Adverse events	Any grade (%)	Grade 3 (%)	Grade 4 (%)
Pruritus	40 (95.2)	0 (0)	0 (0)
Fatigue	38 (90.5)	0 (0)	0 (0)
Elevated transaminases	25 (59.5)	2 (4.8)	1 (2.4)
Diarrhea	21 (50)	1 (2.4)	0 (0)
Rash	18 (42.9)	4 (9.5)	1 (2.4)
Decreased platelet	12 (28.6)	1 (2.4)	0 (0)
Cutaneous toxic effects	11 (26.2)	2 (4.8)	2 (4.8)
Hypothyroidism	5 (11.9)	2 (4.8)	1 (2.4)
Proteinuria	4 (9.5)	1 (2.4)	2 (4.8)
Myocarditis	1 (2.4)	0 (0)	1 (2.4)
Hypoadrenocorticism	1 (2.4)	0 (0)	1 (2.4)

## Discussion

Hepatocellular carcinoma is a commonly occurring cancer with a significant rate of mortality (1). Despite advancements in early detection methods for HCC, the majority of patients are still identified in later stages, leading to unfavorable outcomes. The average overall survival for untreated patients is a mere 4 months (2). Thankfully, several treatments, including immunotherapy, targeted therapy, radiofrequency ablation (RFA), radiation therapy, and TACE, have been innovated and demonstrated to extend HCC patients' survival (16–18).

With the progression of treatment strategies, various external beam radiation therapy (EBRT) approaches have been developed and utilized for locally advanced HCC with favorable outcomes. These modalities include intensity-modulated radiotherapy (IMRT), stereotactic body radiotherapy (SBRT), and gamma knife radiosurgery (GKR) (19–27). EBRT has been proven to decrease the risk of liver failure by safeguarding the adjacent healthy tissue while delivering a concentrated radiation dosage. TACE has also exhibited significant potential as a therapeutic choice for HCC. Multiple investigations have confirmed that TACE is a reliable and secure treatment alternative for HCC as long as the feeding artery of the tumor can be identified. The most recent research indicated that advanced HCC patients treated with TACE + Lenvatinib had an mPFS of 10.6 months and an mOS of 17.8 months (28).

Postoperative adjuvant therapy is an important means to reduce the risk of tumor recurrence and metastasis and improve patient survival. Compared with neoadjuvant therapy, postoperative adjuvant therapy can more accurately select treatment groups and individualized treatment plans based on postoperative pathology and molecular classification, without delaying surgery. The population for postoperative adjuvant therapy is mainly liver cancer patients who are suitable for surgical resection and have a high risk of recurrence and metastasis. Although the high-risk recurrence and metastasis factors defined in different studies are different. Previous studies have shown that the preoperative elevation of alkaline phosphatase and lactate dehydrogenase is closely related to postoperative recurrence (29). In addition, plasma lymphocyte rate (Lym-R) and aspartate aminotransferase (AST) score can predict the prognosis of HCC treated with TACE or TACE combined with systemic therapy (30). Currently recognized high-risk recurrence and metastasis factors evaluated after surgery generally include: tumor rupture, tumor diameter >5 cm, multiple tumors, microvascular invasion (MVI), large vessel invasion, lymph node metastasis, positive resection margin or narrow resection margin (31–33).

Currently, there is no standardized approach for adjuvant therapy following curative resection in HCC patients. Previous studies have shown that postoperative adjuvant transcatheter arterial chemoembolization (PA-TACE) can significantly improve the prognosis of HCC patients with risk factors of recurrence, including PVTT (34). This suggests that postoperative adjuvant antitumor therapy can decrease the risk of recurrence and prolong survival time. Currently, systemic therapy using tyrosine kinase inhibitors (TKIs) and ICIs has become an important part of HCC treatment. Unfortunately, the STORM study demonstrated that postoperative adjuvant sorafenib is not effective for HCC after hepatectomy in patients with high-risk recurrence factors (35). However, there have been significant advancements in the efficacy of anti-PD-1 antibody inhibitors. These inhibitors demonstrate promising outcomes by augmenting the patient's immune response and reinstating its capacity to eradicate malignant cells. In addition, recent research has indicated that the combination of molecular targeted therapy and immunotherapy has exhibited promising results in the treatment and reduction of unresectable HCC patients. Previous studies have demonstrated that the utilization of Lenvatinib and PD-1 inhibitors, as well as TACE triple therapy, can potentially result in positive survival outcomes (36). Examining the impact of the I-O and MTT strategy in postoperative HCC patients, specifically those with high-risk recurrence factors, is of utmost importance given their exceptional performance across various tumor types. The substantial recurrence rate in HCC continues to significantly impact the post-surgical survival (37, 38). Accordingly, it is crucial to administer suitable adjuvant therapy postoperatively to enhance the survival rates of high-risk recurrence patients.

In our study, before 1:1 PSM, there were no statistically significant differences in recurrence and survival rates among patients who received the combination of I-O and MTT compared with patients who did not receive the combination of I-O and MTT. However, after PSM, patients who received the combination of I-O and MTT had significantly lower rates of recurrence and mortality and a better prognosis than those who did not receive I-O and MTT. The findings of Zhang and fellow researchers align closely with this conclusion since they unveiled that utilizing anti-PD-1 antibodies after surgery could significantly enhance the rates of overall survival and recurrence-free survival for HCC patients at high risk of recurrence (39). Compared with their study, our study included a small sample size, and the results were easily affected by individual differences. Moreover, before PSM, there was significant heterogeneity in baseline data between the two groups, such as gender, AFP, tumor size, MVI, satellite foci, tumor number, and tumor envelope, which may be significantly different from the inclusion criteria of the two groups at the beginning of treatment. This is the disadvantage of retrospective study, which also indicates the necessity of PSM. Univariate and multivariate Cox proportional regression analysis was employed to explore the connection between risk factors and survival. It is noteworthy that the correlation of AFP, incomplete tumor capsule and I-O and MTT and overall survival after hepatectomy for HCC with PVTT has been established. These parameters have been validated to influence the outcome of HCC with PVTT. This implyes that I-O and MTT serve as crucial prognostic factors for HCC. Additionally, we further enhanced the nomogram by incorporating I-O and MTT and verified that the model demonstrates excellent predictive capability. Moreover, the AEs linked to I-O and MTT were relatively mild, with only a small number of individuals encountering grade 3–4 AEs, and there were no fatalities ascribed to AEs occurring after treatment. Nonetheless, it is imperative to be attentive to patients receiving specific combined treatments after surgery, such as postoperative TACE. This particular set of patients might be at a higher risk of experiencing grade 3–4 AEs. Hence, meticulous monitoring is indispensable throughout postoperative adjuvant therapy, especially when combination treatments are involved.

There were several limitations to this study. Firstly, the data we collected originated solely from one study center and were obtained retrospectively. To further confirm the impact of I-O and MTT on these patients, it is necessary to carry out a prospective, multicenter, randomized clinical trial. Secondly, as Asian HCC patients have a higher prevalence of hepatitis B virus infection, the use of antiviral therapy after surgery can greatly affect patient outcomes. Finally, HBV was the major etiology. The effect of I-O for non-HBV non-HCV hepatocellular carcinoma (NBNC-HCC) might be decreased. HBV was the major etiology. The effect of I-O for NBNC-HCC might be decreased. Previous studies have shown that compared with non-viral-related HCC, the tumor immune microenvironment of HBV-HCC had a stronger immunosuppressive effect, which was reversed by PD-1 inhibitors (40).

## Data Availability

The datasets presented in this study can be found in online repositories. The names of the repository/repositories and accession number(s) can be found below: https://doi.org/10.6084/m9.figshare.20499261.
